# Genome-Wide Regulation of Acupuncture and Moxibustion on Ulcerative Colitis Rats

**DOI:** 10.1155/2021/9945121

**Published:** 2021-10-08

**Authors:** Zhaoqin Wang, Yan Huang, Di Wang, Rumeng Wang, Kunshan Li, Qin Qi, Zhe Ma, Muen Gu, Handan Zheng, Yuan Lu, Luyi Wu

**Affiliations:** ^1^Department of Aeronautics and Astronautics, Shanghai Key Laboratory of Acupuncture Mechanism and Acupoint Function, Fudan University, Shanghai 200433, China; ^2^Shanghai Research Institute of Acupuncture and Meridian, Shanghai 200030, China; ^3^Yueyang Hospital of Integrated Traditional Chinese and Western Medicine, Shanghai University of Traditional Chinese Medicine, Shanghai 200437, China; ^4^Shanghai TCM-integrated Hospital, Shanghai University of Traditional Chinese Medicine, Shanghai 200082, China; ^5^Shanghai University of Traditional Chinese Medicine, Shanghai 201203, China

## Abstract

Acupuncture and moxibustion have definite clinical effects on treating ulcerative colitis (UC), but their mechanism is still unclear. To investigate the molecular mechanisms, we applied herb-partitioned moxibustion or electroacupuncture at the Tianshu (ST25) points on UC rats and used RNA sequencing to identify molecular consequences. Male Sprague Dawley (SD) rats were divided into 6 groups randomly: the normal control (NC) group, the control + herb-partitioned moxibustion (NCHM) group, the control + electroacupuncture (NCEA) group, the model (UC) group, the model + herb-partitioned moxibustion (UCHM) group, and the model + electroacupuncture (UCEA) group. Compared to the UC group, HE staining in the UCHM group and UCEA group indicated that colitis was relieved, the histopathological score and MPO were both significantly reduced, and the serum hs-CRP concentration was decreased significantly. The results of RNA-seq suggested that, compared to the NC group, 206 upregulated genes and 167 downregulated genes were identified in colon tissues from the UC group; compared to the UC group, the expression levels of some genes were both affected in the UCHM group and the UCEA group (684 differentially expressed genes were identified in the UCHM group, and 1182 differentially expressed genes were identified in the UCEA group). KEGG signal pathway analysis indicated that the differentially expressed genes in the UCHM group were associated with the JAK-STAT signaling pathway and cell adhesion molecule (CAM); the differentially expressed genes in the UCEA group were associated with the NF-*κ*B signaling pathway, the toll-like receptor signaling pathways, the PI3K-Akt signaling pathway, the MAPK signaling pathway, and the Wnt signaling pathway. This is the first study to reveal the gene expression characteristics of the anti-inflammatory effect of UC rats from the perspective of acupuncture and moxibustion control, which provide a clue for further investigation into the molecular mechanisms of UC treatment by acupuncture and moxibustion.

## 1. Introduction

Ulcerative colitis (UC) is characterized by chronic nonspecific inflammation involving the colonic and rectal mucosa. UC is considered an intractable disease in clinical practice, and the etiology and pathogenesis have been incompletely elucidated. The clinical manifestations mainly include abdominal pain, diarrhea, and bloody purulent stool. Most patients experience lifelong recurrence, which causes immense pain and severely influences their quality of life. In recent decades, statistical analyses have shown that the incidence of UC in Europe and North America shows an increasing trend year by year [1]. In China, the incidence of UC is rising sharply in recent years as people's lifestyles have changed and diagnostic levels have improved [2]. The pathogenesis of UC is not yet completely clear, and modern medicine considers that its onset may be associated with many factors, mainly including genetics, environment, diet, gut microbiota, and immunity [3]. The current treatment methods for UC in clinical practice mainly include drug control and surgery; however, both methods have certain limitations. Drugs used to treat UC can only improve symptoms and delay disease progression but cannot effectively control or cure UC, and the long-term therapeutic efficacy is not ideal. In addition, adverse reactions to drugs have caused many problems for patients that further influence the therapeutic efficacy.

Acupuncture and moxibustion have better therapeutic effects on UC, and their simple, practical, inexpensive, and convenient features are accepted by some patients. We have carried out many clinical trials and animal experiments, and the results indicate that acupuncture and moxibustion have excellent therapeutic effects on UC [4–7]. Joos et al. performed randomized controlled studies to observe the therapeutic effects of acupuncture and moxibustion on UC patients and confirmed that the acupuncture and moxibustion procedures of traditional Chinese medicine (TCM) had certain efficacy in the treatment of UC [8]. Acupuncture and moxibustion have advantages for controlling disease activity in UC patients, improving patients' quality of life, maintaining clinical remission, and preventing and treating complications [9–12]. However, the effect of acupuncture and moxibustion on the regulation of colon tissue transcriptome remains unknown. There is an urgent need to reveal the commonalities and differences between acupuncture and moxibustion in the molecular mechanism of anti-inflammation in UC. Therefore, we applied acupuncture and moxibustion in UC rats to evaluate the anti-inflammatory effects of acupuncture and moxibustion and explored the mechanism of acupuncture and moxibustion at the molecular level of the transcriptome by RNA sequencing. Our data suggest the difference in gene expression and signaling pathways between each group, which contribute to our knowledge of the effect of acupuncture and moxibustion on UC rats at the molecular level.

## 2. Materials and Methods

### 2.1. Experimental Animals

We carried out all experimental procedures in strict accordance with the regulations of “Instructive Notions with Respect to Caring for Laboratory Animals” released by the Ministry of Science and Technology, China. The animal certificate number is 2015000516185. Any rats that died during surgery were excluded from the study. Healthy adult male Sprague Dawley (SD) rats with a bodyweight of 180 ± 200g were purchased from Shanghai SLAC Laboratory Animal Co., Ltd, and the license number was SCXK (Beijing) 2012–0001. All animals were maintained in cages under a 12/12-h light/dark cycle at 20 ± 2°C and a controlled relative humidity of 50%–70%, and they were allowed free access to food and water for one week.

### 2.2. Model Establishment

A total of 66 normal rats were used in this study, 33 of which were randomly selected for model establishment. After 1 w of adaptive feeding, the SD rats received 4% dextran sulfate sodium (DSS, MP biomedicals, No. 0216011080) in water for 7 d, followed by 1% DSS in water for another 7 d [13]. The bodyweight and overall conditions of the rats were recorded daily. After that, the model of UC rats was identified. The colonic mucosa of UC rats showed large ulcer formation, abnormal morphology of glands, or even heterogeneous hyperplasia, and disorderly arrangement, a large number of inflammatory cells infiltrating the mucosa and submucosa, congestion, and edema were seen, which confirmed the successful establishment of the UC rat model [14].

### 2.3. Grouping and Treatment

**Figure 1:**
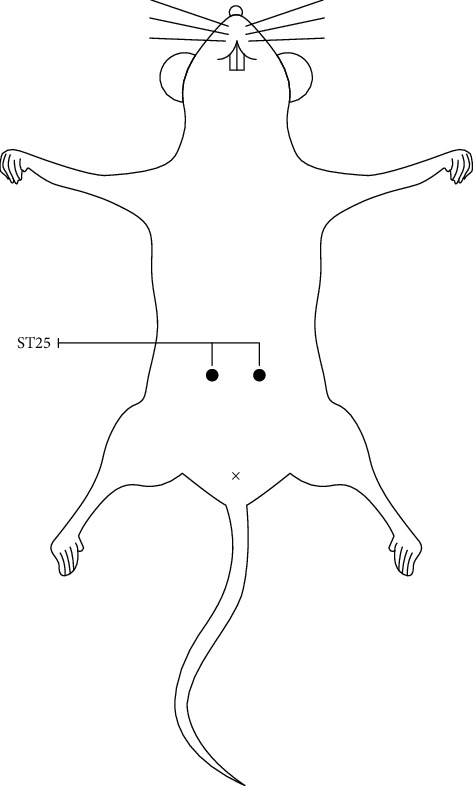
Location of ST25 of a rat.

Rats that met the study requirements were randomly selected for model establishment. UC model establishment was evaluated by comparing three model rats with three normal rats. In the experiment, 30 rats were randomized to the normal control (NC) group, the control + herb-partitioned moxibustion (NCHM) group, and the control + electroacupuncture (NCEA) group, 10 rats in each group. A total of 30 rats were studied, which were randomly divided into the model (UC) group, the model + herb-partitioned moxibustion (UCHM) group, and the model + electroacupuncture (UCEA) group, 10 rats in each group. In the NC and UC groups, the rats did not receive any treatment but underwent the same manipulation and immobilization as in the treatment groups. In the NCHM group and UCHM group, the rats received herb-partitioned moxibustion at the ST25 acupoints (bilateral). Based on an anatomic method referenced in the “Map of Animal Acupoints” from Shi Yan Zhen Jiu Xue written by Lin WZ, ST25 is located on the abdomen of the rat, level with the umbilicus and 5 mm next to it (Figure 1). In the NCEA group and UCEA group, the rats received electroacupuncture at the ST25 acupoints (bilateral).

### 2.4. Herb-Partitioned Moxibustion and Electroacupuncture Interventions

#### 2.4.1. Herb-Partitioned Moxibustion

The rats were immobilized with custom-made rat fixators. The weight of the moxa cone was approximately 90 mg. The medicinal cake contained aconite and cinnamon and was mixed and stirred with yellow wine to form a thick paste. The medicinal cakes were prepared using a mold with a thickness of approximately 0.5 cm and a diameter of 1 cm. The medicinal cakes were placed at the bilateral ST25 acupoints. Each acupoint received two moxa cones each time (about 10 mins in total). Moxibustion was performed once daily for a total of seven treatments.

#### 2.4.2. Electroacupuncture

The rats were immobilized with custom-made rat fixators. Acupuncture needles were vertically inserted (2 mm) into the bilateral ST25 acupoints. The needle handle was connected to Han's acupoint nerve stimulator (Hans-200). An electroacupuncture needle with a frequency of 2/100 Hz and a current of 1 mA was used. Electroacupuncture was performed once daily at the same time for 10 min for a total of seven treatments.

In the control group and the model group, all rats were immobilized with custom-made rat fixators for 10 min once daily.

### 2.5. Specimen Collection and Processing

One week after the end of the intervention, the rats were anesthetized by an intraperitoneal injection of 2% pentobarbital sodium (40–50 mg/kg). After anesthesia induction, the abdominal cavity of each rat was opened, and a 4 cm segment of the colon 2 cm above the anus was collected. The colon was cut open longitudinally along the mesentery to observe the gross condition of the colon and perform macroscopic injury scoring. Finally, a 3 cm segment of the colon from the bottom to the top was collected and divided into three pieces. One piece was fixed in a 10% neutral formalin solution and stored for future use, and the other two pieces were minced, mixed, and divided into two copies, which were both placed in cryotubes and temporarily stored in liquid nitrogen. After the material collection was complete, the copies were transferred to a -80 °C freezer for storage and future use.

### 2.6. Pathological Observations of Rat Colon Tissues

The rat colon samples fixed in 10% neutral formalin solution were trimmed to an appropriate size, dehydrated, embedded, and prepared into 4 µm thick sections. After the sections were stained with hematoxylin-eosin (HE), colonic mucosal epithelia and colonic crypts were observed under a light microscope to identify inflammatory cell infiltration or proliferation of granulation tissue. Histopathological colon injury scoring was performed with reference to the method of Butzner et al. [15].

### 2.7. ELISA Detection

The concentration of high-sensitivity C-reactive protein (hs-CRP) in serum and the enzyme activity of myeloperoxidase (MPO) in colon tissue were detected using ELISA. Six rat samples from each group were randomly selected for detection. The specific manipulation procedure is described below. A 96-well microplate was used, and two replicate wells were set up. Standards were sequentially added to standard wells at 50 *µ*l/well. Except for blank wells, the samples to be measured were sequentially added to the sample wells at 10 *µ*l/well, and then a diluted sample solution was added at 40 *µ*l/well and evenly mixed. Horseradish peroxidase-labeled antibody was added to the standard wells and sample wells at 100 *µ*l/well but not the blank wells. The plate was incubated in a 37°C incubator for 60 min. The plate was washed five times and completely dried on a filter paper. Substrates A and B were sequentially added to each well at 50 µl/well, followed by incubation in a 37°C incubator in the dark for 15 min. The stop solution was added at 50 *µ*l/well, and the optical density (OD) value (450 nm wavelength) of each well was measured within 15 min using a microplate reader.

### 2.8. RNA High-Throughput Sequencing

In the NC group, UC group, UCHM group, and UCEA group, colon tissues from 3 rats in each group were randomly collected, and total RNA was extracted using the Trizol-based method. Quality control of the extracted total RNA was performed using the Agilent 2200. Libraries were constructed, and library quality was examined using the Agilent 2200. Finally, high-throughput sequencing of the extracted samples was performed using the HiSeq 4000 sequencing platform. The data obtained from sequencing were filtered, subjected to strict quality control, and analyzed using bioinformatics.

### 2.9. RT-qPCR

32 RNA samples from HC, UC, UCHM, and UCEA (8 samples each group) were examined with RT-qPCR. 1 *μ*g total RNA from each sample was reverse transcribed into cDNA using SYBR ® Green. All RT-qPCR assays were performed in duplicate. Reactions were carried out with SYBR *R* Premix Ex TaqTM kit (TaKaRa) and performed on Light Cycler ® 480 II System (Roche). For comparison of relative gene expression, we analyzed RT-qPCR data by the ΔCt method and normalized the value to endogenous GAPDH control.

### 2.10. Statistical Analysis

The quantitative data are presented as the mean ± standard error of the mean (SEM) of at least three experimental repeats. Data are presented as mean ± SEM. SPSS 21.0 statistical software was used for all statistical analyses, and GraphPad Prism (GraphPad, San Diego, CA, USA) was used for mapping. The significance of the differences between groups was evaluated using Student's *t*-test for two groups and one-way analysis of variance (ANOVA) for multiple-group comparison. Kruskal–Wallis H was used for the distribution of ranked data. The statistical levels were all *α* = 0.05, and *P* < 0.05 indicated that the difference was statistically significant.

## 3. Results

### 3.1. Observation of the Effect of Acupuncture and Moxibustion on UC Rats

#### 3.1.1. Histopathological Observation of Colon Tissues from Rats in All Groups

**Figure 2:**
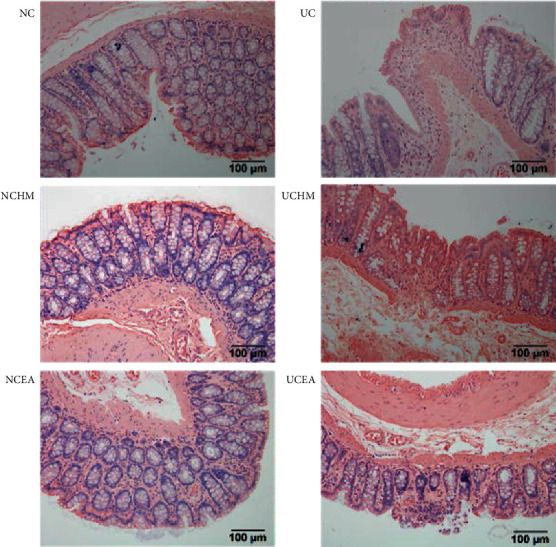
Histopathological observation of the colon. NC: normal control group; UC: model group; NCHM: control + herb-partitioned moxibustion group; UCHM: model + herb-partitioned moxibustion group; NCEA: control + electroacupuncture group; UCEA: model + electroacupuncture group.

In the rats in the NC group, NCHM group, and NCEA group, the mucous membrane of the colon was covered with intact epithelia, no ulcers were observed, the structures of all layers of tissues were clear, the crypts exhibited an orderly arrangement, only a small amount of inflammatory cell infiltration was noted in the mucosal layer, and no interstitial congestion or edema was evident. In the UC group, the epithelia covering the surface of the colon had fallen off, the number of crypts in the mucosal layer was decreased, large numbers of lymphocytes, plasma cells, neutrophils, and histiocytes had infiltrated the interstitial and submucosal layers, microvascular proliferation was evident, and lymphoid follicle formation was observed in some samples. Compared to the UC group, the number of inflammatory cells in rat colon tissues was significantly decreased, the tissue structure was normal, and healing ulcers were noted in the UCHM and UCEA groups (Figure 2).

**Figure 3:**
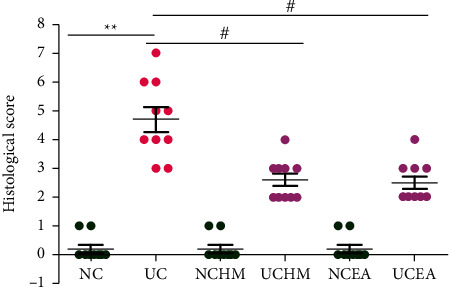
Histopathological colon injury score in each group. Compared with the NC group, ^*∗*^*P* < 0.01; compared with the UC group, ^#^*P* < 0.01 (*n* = 10).

The histopathological colon injury score was significantly higher in the UC group than that in the NC group (*P* < 0.01). The histopathological colon injury scores were significantly lower in the UCHM group and UCEA group than those in the UC group (both *P* < 0.01) (Figure 3).

#### 3.1.2. Results of Serological Indicator Detection

**Figure 4:**
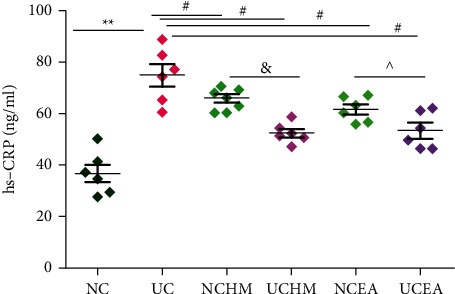
Serum hs-CRP concentration in each group. Compared to the NC group, ^*∗*^*P* < 0.01; compared to the UC group, ^#^*P* < 0.01; compared to the NCHM group, ^&^*P* < 0.05; compared to the NCEA group, ^*P* < 0.05 (*n* = 6).

The serum hs-CRP concentration in the rats in the UC group was significantly higher than that in the NC group (*P* < 0.01). The serum hs-CRP concentrations in the rats in the UCHM group and UCEA group were significantly lower than those in the UC group (*P* < 0.01). The serum hs-CRP concentration in the rats in the UCHM group was significantly lower than that in the UC group (*P* < 0.05). The serum hs-CRP concentration in the rats in the UCEA group was significantly lower than that in the UC group (*P* < 0.05) (Figure 4).

#### 3.1.3. Results of Enzyme Activity of Myeloperoxidase Detection in Colon Tissues

**Figure 5:**
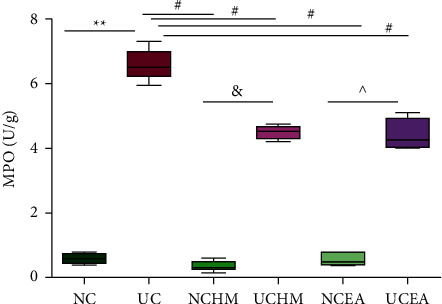
MPO concentration in each group. Compared to the NC group, ^*∗*^*P* < 0.01; compared to the UC group, ^#^*P* < 0.01; compared to the NCHM group, ^&^*P* < 0.05; compared to the NCEA group, ^*P* < 0.05 (*n* = 6).

The MPO concentration in rat colon tissues in the UC group was significantly higher than that in the NC group (*P* < 0.01). The MPO concentrations in rat colon tissues in the NCHM group, NCEA group, UCHM group, and UCEA group were significantly lower than those in the UC group (all *P* < 0.01). The MPO concentration in rat colon tissues in the NCHM group was significantly lower than that in the UCHM group (*P* < 0.05). The MPO concentration in rat colon tissues in the NCEA group was significantly lower than that in the UCEA group (*P* < 0.05) (Figure 5).

### 3.2. Effects of Acupuncture and Moxibustion on Gene Expression Profiles in Colon Tissues from UC Rats

#### 3.2.1. The Differentially Expressed Gene Expression Profiles in All Groups

The colon tissues of three rats each from the NC group, UC group, UCHM group, and UCEA group were collected for extraction of total RNA. The RNA-seq sequencing method was performed to observe the effects of acupuncture and moxibustion on gene expression profiles in rat colon tissues. Compared with the NC group, 373 differentially expressed genes were identified in colon tissues from the UC group, including 206 upregulated genes and 167 downregulated genes. Compared with the UC group, 684 differentially expressed genes were identified in the UCHM group, including 380 upregulated genes and 304 downregulated genes (for top 10 regulated genes, see Tables 1 and 2), and 1182 differentially expressed genes were identified in the UCEA group, including 720 upregulated genes and 462 downregulated genes (for top 10 regulated genes, see Tables 3 and 4).

#### 3.2.2. Overlap of Differentially Expressed Genes between Groups

**Figure 6:**
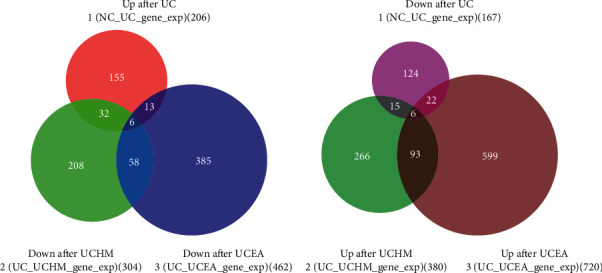
Number of overlapping differentially expressed genes between groups. (a) Overlap of differentially expressed genes in Up after UC, Down after UCHM, and Down after UCEA. (b) Overlap of differentially expressed genes in Down after UC, Up after UCHM, and Up after UCEA.

Overlapping differentially expressed genes were analyzed and screened using the Venn diagram. Compared to the NC group, DSS-induced UC colon tissues had 206 upregulated genes, 38 of which overlapped with 304 downregulated differentially expressed genes in the UCHM group, while 19 differentially expressed genes overlapped with 462 downregulated genes in the UCEA group. In addition, a total of six overlapped genes were identified among these three groups (Figure 6(a)). Furthermore, compared to the NC group, 167 downregulated genes were identified in DSS-induced UC colon tissues, 21 of which overlapped with 380 upregulated genes in the UCHM group, while 28 genes overlapped with 720 upregulated genes in the UCEA group. In addition, a total of six overlapped genes were identified among these three groups (Figure 6(b)).

#### 3.2.3. Cluster Analysis of Differentially Expressed Genes in All Groups

**Figure 7:**
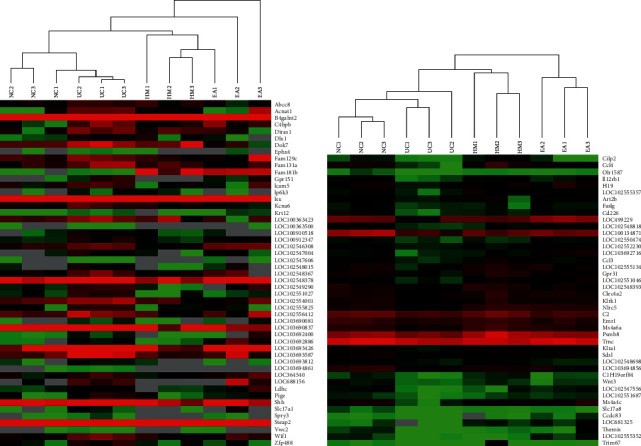
Heatmap of upregulated and downregulated differentially expressed genes. (a) Upregulated differentially expressed genes in UC. (b) Downregulated differentially expressed genes in UC. Bright red represents the highest expression, and the expression decreased with the darkening of red; black represents the expression of 0; dark green to bright green represents the decreased expression.

Cluster and heatmap analyses of differentially expressed mRNAs were performed using Cluster 3.0 and Tree view software to intuitively compare uniformity and differences in samples among the groups. Hierarchical cluster analysis was performed on a total of 12 samples from four groups to analyze 51 upregulated differentially expressed genes and 43 downregulated differentially expressed genes in the UC group that overlapped with genes in other groups. The results suggested that the genes in the UC group were all clustered together. In addition, the genes in the UCHM group and the UCEA group were also clustered together. The expression levels in three samples from each group were more consistent, a significant difference was found between the NC group and the UC group, and a significant difference was also found among the UCHM group, UCEA group, and UC group. Genes that were upregulated in the UC group were downregulated in the UCHM group and UCEA group, similar to observations in the NC group. In contrast, the results were the same (Figure 7).

#### 3.2.4. Gene Ontology (GO) and Pathway Analyses of Differentially Expressed Genes in All Groups

**Figure 8:**
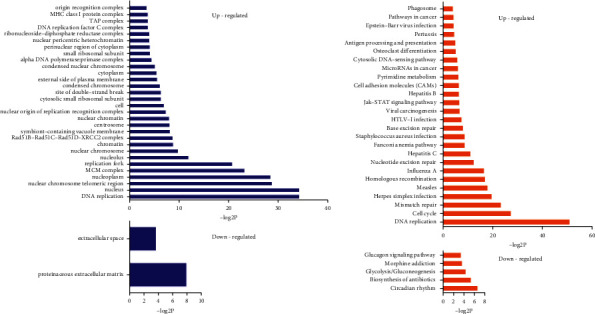
(a) GO analyses and (b) pathway analyses of differentially expressed genes of the UCHM group compared with the UC group.

The results of GO analyses of differentially expressed genes in colon tissues in the UCHM group suggested that the GO terms associated with 380 upregulated genes were mainly associated with biological functions of the nucleus, chromosome, and telomere regions, chromatin, and the major histocompatibility complex (MHC) class I protein complex. Only two GO terms were associated with downregulated genes, i.e., protein extracellular matrix and extracellular space (Figure 8(a)). Pathways that were associated with upregulated genes included DNA repair, the cell cycle, nucleotide excision repair, the JAK-STAT signaling pathway, cell adhesion molecule (CAM), miRNA function in cancer, and the phagosome. Furthermore, downregulated genes were closely associated with circadian rhythms, biosynthesis of antibiotics, and glycolysis/gluconeogenesis (Figure 8(b)).

**Figure 9:**
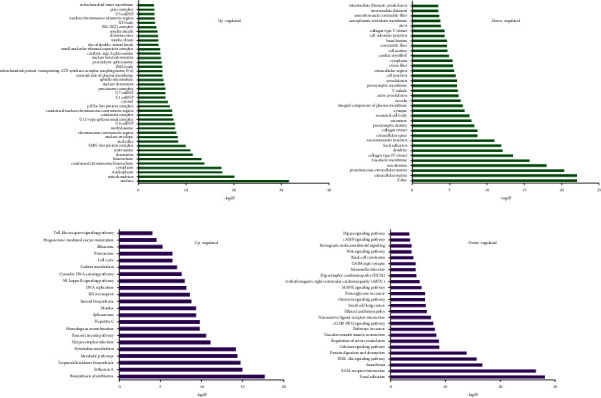
(a) GO analyses and (b) pathway analyses of differentially expressed genes of the UCEA group compared with the UC group.

The GO analysis results of differentially expressed genes in colon tissues in the UCEA group suggested that the GO terms associated with 720 upregulated genes were mainly associated with the nucleus, chromatin, chromosomes, the SMN-Sm protein complex, and mitochondrial proton transport. The GO terms associated with 380 downregulated genes were mainly closely associated with biological functions of the protein extracellular matrix, muscle fiber, basement membrane, collagen type IV (trimmer), postsynaptic density, neuronal cell body, synapses, cell junction, basal layer, and contractile fibers in smooth muscle (Figure 9(a)). Pathways that were associated with upregulated genes were mainly associated with the biosynthesis of antibiotics, terpenoid skeleton biosynthesis, metabolic pathways, pyrimidine metabolism, steroid biosynthesis, RNA transport, DNA replication, the NF-*κ*B signaling pathway, cytosolic DNA-sensing pathway, carbon metabolism, the cell cycle, the proteasome, the ribosome, and Toll-like receptor signaling pathways. Pathways that were associated with downregulated genes were mainly associated with adhesion, ECM-receptor interaction, the PI3K-Akt signaling pathway, protein digestion and absorption, the calcium signaling pathway, regulation of the actin cytoskeleton, vascular smooth muscle contraction, cancer pathways, cGMP-PKG signaling pathway, neuroactive ligand-receptor interaction, proteoglycan in cancer, the MAPK signaling pathway, the GABAergic synapse, the Wnt signaling pathway, and the cAMP signaling pathway (Figure 9(b)).

### 3.3. Validation of DEGs with RT-qPCR

**Figure 10:**
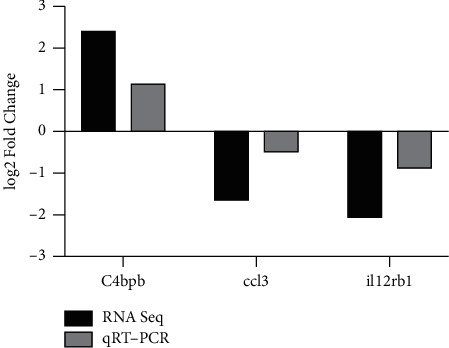
Validation of selected DEGs with RT-qPCR. The statistical graph showed the fold change (log2) of the mRNA expression of DEGs by RNA-seq (black) and RT-qPCR (grey), normalized to the GAPDH level.

To verify the reliability of RNA-seq results, 8 transcriptomic data were randomly selected from each group, and 3 DEGs expressions were tested following the results of RNA-seq data: c4bpb, ccl3, and il12rb1. It can be seen in Figure 10 that the RT-qPCR results of the three genes were consistent with RNA-seq analysis. The identification demonstrated the reliability of our RNA-seq results (Figure 10).

## 4. Discussion

The ST25 acupoint is an acupuncture point of the Stomach Meridian of Foot-Yangming. This acupoint is the front-mu point of the large intestine and mainly dredges and regulates the intestine. The ST25 acupoint was regarded as the key acupoint for the treatment of abdominal distension and diarrhea in ancient times. Many modern clinical studies have shown that the ST25 acupoint has excellent bidirectional regulatory effects on gastric and intestinal functions and significant efficacy in the treatment of gastric and intestinal diseases such as abdominal pain, diarrhea, constipation, and dysentery [16–18].

In this study, we found that the general condition of the rats with DSS-induced UC gene gradually improved after herb-partitioned moxibustion and electroacupuncture interventions. Microscopic scoring of colon tissue injury showed that colon tissues from DSS-induced UC rats exhibited obvious injury. After herb-partitioned moxibustion and electroacupuncture interventions, the UC rat tissues exhibited mild injury, less bleeding, and less feculent matter. In addition, histopathological features of the colon tissues included ulcer surface healing, mucosal epithelial hyperplasia and covering, and less lymphocyte and plasma cell infiltration. These results suggested that herb-partitioned moxibustion and electroacupuncture had protective effects on UC rat colon against DSS-induced injury.

The CRP level has an obvious correlation with the Mayo score of UC disease and moderate positive correlations with both the clinical classification and endoscopic classification of UC [19, 20]. Detection of the serum CRP level can objectively reflect changes in inflammation and disease conditions in UC patients and can be used as an indicator of colonic mucosal repair. In this study, the hs-CRP concentration in UC rats was higher than that in the NC group, indicating a large increase in CRP in the serum in UC inflammatory conditions; however, herb-partitioned moxibustion and electroacupuncture both significantly decreased the CRP level in rats with DSS-induced UC, thus controlling the inflammation in the colon. MPO activity in the colon shows a linear correlation with the level of neutrophil infiltration in tissues [21] and is a reliable indicator for evaluating the levels of neutrophil infiltration and inflammation in tissues [22]. An increased MPO level can promote the proliferation and activation of inflammatory effector cells, and effector cells more easily penetrate endothelial barriers to reach local inflammatory tissues and cause inflammation in the colon and the formation of ulcers [23]. This study showed that the MPO level in colon tissues from UC rats was significantly higher than that in the NC group rats, indicating that the level of neutrophil infiltration in colon tissues from UC rats was more severe, which is consistent with the histopathological results. The MPO levels in the UCHM and UCEA groups were both lower than those in the UC group, indicating that herb-partitioned moxibustion and electroacupuncture interventions can both effectively reduce the level of neutrophil infiltration in UC rats and reduce inflammatory responses in the colon.

The development of UC is considered caused by the joint actions of many factors, with genetic factors playing an important role. UC has been shown to exhibit the familial aggregation phenomenon. The risk of UC in family members of patients is significantly higher than that in the non-UC population [24]. To further elucidate the genetic features of UC and the specific mechanism underlying the effects of acupuncture and moxibustion in the treatment of UC, this study observed the effects of herb-partitioned moxibustion and electroacupuncture on gene expression profiles in colon tissues from UC model rats from a transcriptome perspective. This study showed that, compared to the NC group, the rats in the UC group had 373 differentially expressed genes, including 206 upregulated genes and 167 downregulated genes. Herb-partitioned moxibustion and electroacupuncture both affected gene expression profiles in colon tissues and could upregulate or downregulate the expression of some genes. The number of upregulated differentially expressed genes was higher than the number of downregulated differentially expressed genes. The heatmap and cluster analyses of differentially expressed genes in all groups showed a significant difference between the NC group and the UC group. The UCHM and UCEA groups also showed significant differences compared to the UC group. In addition, the upregulated genes in the UC group showed a trend of downregulation in the UCHM and UCEA groups similar to that in the NC group. Genes that were downregulated in the UC group showed a trend of upregulation in the UCHM and UCEA groups. The above results indicated that herb-partitioned moxibustion and electroacupuncture had a positive regulatory effect on gene expression profiles in colon tissues from UC rats and could orient them in a normal direction.

Furthermore, we performed GO and pathway functional enrichment analyses on differentially expressed genes. The results showed that 380 upregulated genes in the UCHM group were associated with many biological functions, including the nucleus, chromosome, and telomere regions, chromatin, and the MHC class I protein complex, the latter of which should receive special attention. MHC molecules participate in antigen presentation and are necessary for the differentiation and maturation of T cell immunity. They play important roles in the initiation and regulation of immune responses. MHC molecules are closely associated with immune responses, immune regulation, and the production of some pathological conditions in the body [25]. MHC class I molecules process and present endogenous antigens through the cytosolic pathway. At the gene level, the results confirmed that treatment of UC by herb-partitioned moxibustion was closely associated with enhanced immune responses and immune regulatory functions in the body. The GO terms associated with downregulated genes in the UCHM group included the protein extracellular matrix and extracellular space, which were associated with the colon tissue structure and cellular physiological activities of rats. The biological functions associated with upregulated genes in the UCEA group were similar to those in the UCHM group. The GO terms associated with downregulated genes included protein extracellular matrix, muscle fiber, postsynaptic density, neuronal cell body, synapses, and cell junctions, which were mainly associated with the colon tissue structure and neuronal activities of rats. These differentially expressed genes regulated by herb-partitioned moxibustion and electroacupuncture are mainly involved in many signaling pathways related to inflammation and immune response, revealing to some extent the differential pathways of the anti-inflammatory effects of herb-partitioned moxibustion and electroacupuncture. Herb-partitioned moxibustion mainly regulates the expression of the JAK-STAT signaling pathway, cell adhesion molecule (CAM) related genes, while electroacupuncture can regulate NF-*κ*B signaling pathway, the Toll-like receiver signaling pathways, the PI3K-Akt signaling pathway, the MAPK signaling pathway, and the Wnt signaling pathway. The IL and TNF superfamilies and many cytokines, including interferon, all participate in the development and progression of intestinal inflammation, and the above signal transduction pathways are closely associated with the regulation and release of these cytokines [26–28]. This study showed that electroacupuncture can downregulate many genes associated with the PI3K-Akt and Wnt signaling pathways. Previous studies have indicated that the PI3K-Akt signaling pathway mediates the development of UC by promoting inflammation and activating T cells [29]. In addition, abnormal activation of the Wnt signaling pathway was closely associated with canceration of UC [30, 31]. Modern experimental studies have indicated that acupuncture and moxibustion have obvious advantages for the adjustment of immune functions in the body [32], which is mainly achieved through the regulation of immune molecules and immune cells. The immune molecules regulated by acupuncture and moxibustion mainly include immunoglobulins, cytokines, and complements. The regulated immune cells mainly include neutrophils, red blood cells, T cells, macrophages, and natural killer cells [33–36]. Acupuncture and moxibustion can adjust immune system functions in the body, which is beneficial and bidirectional. Therefore, acupuncture and moxibustion may adjust disordered immune functions to normal status and enhance the antidisease ability of the body.

## 5. Conclusions

In summary, our study indicates that acupuncture and moxibustion at ST25 exerted the effects of anti-inflammation on UC rats and reversed the phenotypes by modulating abnormal gene profiles in the UC. Our results suggest that the anti-inflammatory effects of acupuncture and moxibustion may be achieved by pursuing multiple targets, which provide a clue for further investigation into the molecular mechanisms. We will provide more experimental evidence for the anti-inflammatory of acupuncture through further exploration in our next study.

## Figures and Tables

**Table 1:** The top 10 downregulated genes in UCHM (compared with UC).

Gene name	Description	log2 (FC)	*P*
B4galnt2	*β*-1,4-N-Acetyl-galactosaminyltransferase 2	−4.2020411	<0.001
LOC103693426	Uncharacterized	−3.4374964	<0.001
Bpi	Bactericidal permeability increasing protein	−2.6558263	<0.001
Ildr2	Immunoglobulin-like domain-containing receptor 2	−2.2011603	<0.001
LOC102552725	Uncharacterized	−2.1239904	<0.001
Isx	Intestine-specific homeobox	−2.1092788	<0.001
Nfil3	Nuclear factor, interleukin-3 regulated	−2.0201916	<0.001
Aldob	Aldolase, fructose-bisphosphate B	−1.8416709	<0.001
LOC103693587	Uncharacterized	−1.6990522	<0.001
Acsm3	Acyl-CoA synthetase medium-chain family member 3	−1.6681228	<0.001

FC: fold change; UC: model group; NCHM: control + herb-partitioned moxibustion group.

**Table 2:** The top 10 upregulated genes in UCHM (compared with UC).

Gene name	Description	log2 (FC)	*P*
Ido1	Indoleamine 2,3-dioxygenase 1	7.028188	<0.001
RGD1305184	Similar to CDNA sequence BC023105	5.88236646	<0.001
LOC100909911	Uncharacterized	5.22976595	<0.001
LOC102556989	Uncharacterized	4.52584984	<0.001
Krt86	Keratin 86	3.90703757	<0.001
Cxcl10	C-X-C motif chemokine ligand 10	3.89489776	<0.001
Dbp	D-box binding PAR bZIP transcription factor	3.20964656	<0.001
MGC105567	Similar to cDNA sequence BC023105	3.1181265	<0.001
Igtp	Interferon-gamma-induced GTPase	3.00203997	<0.001
LOC681325	Uncharacterized	2.94009072	<0.001

FC: fold change; UC: model group; NCHM: control + herb-partitioned moxibustion group.

**Table 3:** The top 10 downregulated genes in UCEA (compared with UC).

Gene name	Description	log2 (FC)	*P*
Retnlg	Resistin-like gamma	−4.99819	<0.001
Pkd1l1	Polycystin 1 like 1, transient receptor potential channel interacting	−4.913304	<0.001
Rps10l1	Ribosomal protein S10-like 1	−4.6248477	<0.001
LOC100361240	Uncharacterized	−4.2525595	<0.001
Scgb1a1	Secretoglobin family 1A member 1	−3.2828908	<0.001
LOC102551811	Uncharacterized	−3.258879	<0.001
Crp	C-reactive protein	−3.0156217	<0.001
LOC102556038	Uncharacterized	−2.5977539	<0.001
Hoxd13	Homeobox D13	−2.5781291	<0.001
Evx2	Even-skipped homeobox 2	−2.539294	<0.001

FC: fold change; UC: model group; UCEA: model + electroacupuncture group.

**Table 4:** The top 10 upregulated genes in UCEA (compared with UC).

Gene name	Description	log2 (FC)	*P*
Ly49s6	Ly49 stimulatory receptor 6	5.56793974	<0.001
Klrd1	Killer cell lectin-like receptor D1	5.11232751	<0.001
Ccl4	C-C motif chemokine ligand 4	3.61234831	<0.001
Cd8b	CD8b molecule	3.1351152	<0.001
Phf11	PHD finger protein 11	2.95457726	<0.001
Lag3	Lymphocyte activating 3	2.93189218	<0.001
Cd8a	CD8a molecule	2.9117268	<0.001
Cd3e	CD3e molecule	2.90182201	<0.001
Gzma	Granzyme A	2.81778043	<0.001
LOC103693070	Uncharacterized	2.71738337	<0.001

FC: fold change; UC: model group; UCEA: model + electroacupuncture group.

## Data Availability

The research data used to support the findings of this study are included within the article.
